# Experiential learning to advocacy: A peer-led approach to safe medication disposal in pharmacy education

**DOI:** 10.1371/journal.pone.0343961

**Published:** 2026-04-20

**Authors:** Jia Jia Lee, Li Ling Yeap, Ali Haider Mohammed, Chuan Sheng Yap, Bassam Abdul Rasool Hassan, Juman Abdulelah Dujaili, Pui San Saw, Ali Qais Blebil

**Affiliations:** 1 School of Pharmacy, Monash University Malaysia, Subang Jaya, Selangor, Malaysia; 2 Department of Pharmacy, Al Rafidain University, Baghdad, Iraq; 3 Swansea University Medical School, Swansea University, Swansea, United Kingdom; Pharmaceutical Services Division, MALAYSIA

## Abstract

**Introduction:**

Safe medication disposal is crucial for public health and environmental sustainability, yet public awareness remains limited. While educational campaigns exist, pharmacy students can serve as key advocates in promoting proper disposal practices. This study integrates experiential learning and peer-led education to enhance students’ advocacy skills.

**Methods:**

A two-phase mixed-method approach was used. In Phase 1, 35 pharmacy student ambassadors underwent hands-on training at community pharmacies, actively participating in medication take-back programs. Their experiences were documented and analyzed thematically. In Phase 2, they conducted a peer-led workshop for 43 undergraduate pharmacy students. A pre- and post-test assessed knowledge improvement among workshop participants.

**Results:**

The thematic analysis identified five key themes: increased awareness, recognition of environmental and health risks, sense of responsibility, motivation for advocacy, and challenges in promoting safe disposal. Knowledge among workshop participants significantly improved, increasing from mean score 6.86 to 8.44 (Z = −4.827, p < 0.001), demonstrating the effectiveness of peer-led education.

**Conclusion:**

Experiential learning combined with peer-led education effectively strengthened pharmacy students’ knowledge and advocacy for safe medication disposal. Engaging students as educators not only enhanced their own competencies but also improved awareness among peers. This approach empowers future pharmacists to advocate for responsible disposal practices within their communities.

## Introduction

Safe medication disposal is a critical yet often overlooked aspect of healthcare, with significant implications for public health, environmental protection, and community well-being. Improper disposal practices, such as flushing medications down the toilet or discarding them in household waste, can lead to water contamination, ecological disruption, and inadvertent human exposure to pharmaceuticals [1–4]. While medication take-back programs have been introduced to mitigate these risks, public awareness and participation remain limited. Addressing this issue requires a comprehensive strategy that not only educates the public but also empowers healthcare professionals to advocate for proper disposal practice.

Healthcare professionals play a pivotal role in promoting safe medication disposal, as they influence both clinical practice and public behaviour [5]. Among them, pharmacists are uniquely positioned due to their expertise in medicine management. As future healthcare providers, pharmacy students must therefore be equipped with the necessary knowledge and skills, given their potential to influence public behaviour on this issue [6–8]. Educational interventions have been shown to enhance pharmacist and pharmacy students’ understanding, attitudes, and practices related to medication disposal while fostering a more responsible approach to healthcare delivery [6,9,10]. However, evidence consistently highlights gaps in pharmacy curricula worldwide. For instance, a study among Saudi Arabian pharmacy students reported inappropriate knowledge and practices regarding medication storage and disposal, prompting calls for curriculum reform [11,12]. In Kosovo, Shuleta-Qehaja and Kelmendi found that although students recognised the environmental and public health risks of improper disposal, they lacked awareness of medication return procedures, underscoring the need for structured lectures and workshops on safe disposal within pharmacy curricula [12]. In Ethiopia, pharmacy students demonstrated positive attitudes towards ecopharmacology but inadequate knowledge and poor disposal practices, underscoring the need for education on pharmaceutical pollution within pharmacy programs. Collectively, these findings emphasise that pharmacy curricula often provide little education on medication disposal regulation and environmental impacts [13]. Addressing these gaps requires innovative educational strategies that extend beyond traditional didactic methods.

Beyond conventional education, experiential learning, which engages students through hands-on and real-world applications, has been recognised as a powerful learning method for reinforcing concepts and building confidence [14,15]. Additionally, the impact of experiential learning can be further amplified through peer-led education, where students strengthen their own understanding by teaching their peers. Research shows that peer-assisted learning benefits both tutors and tutees by fostering knowledge exchange, improving intrinsic motivation and developing leadership skills [16]. Integrating both approaches of experiential learning and peer-led education, has the potential to create a sustainable model for knowledge dissemination and advocacy training.

This study evaluates the effectiveness of a student-led approach in bridging the educational gap in safe medication disposal through the integration of experiential learning with peer-led knowledge sharing. By engaging students in advocacy activities and encouraging peer-based dissemination of knowledge, this approach seeks to empower pharmacy students with the skills and motivation to promote proper medication disposal in their future professional roles. It is anticipated that this model will contribute to sustainable education through a cascading effect, foster community involvement, and support the development of a more resilient and environmentally sustainable healthcare system.

### Framework

This study integrates Kolb’s Experiential Learning Cycle (ELC) and the Peer Education Framework to guide the design, implementation and analysis of education intervention on safe medication disposal [15]. Kolb’s theory is well-suited for skill-based and behavioural education, as it emphasizes active participation, allowing learners to internalise concepts more effectively than passive instruction.

**Fig 1:**
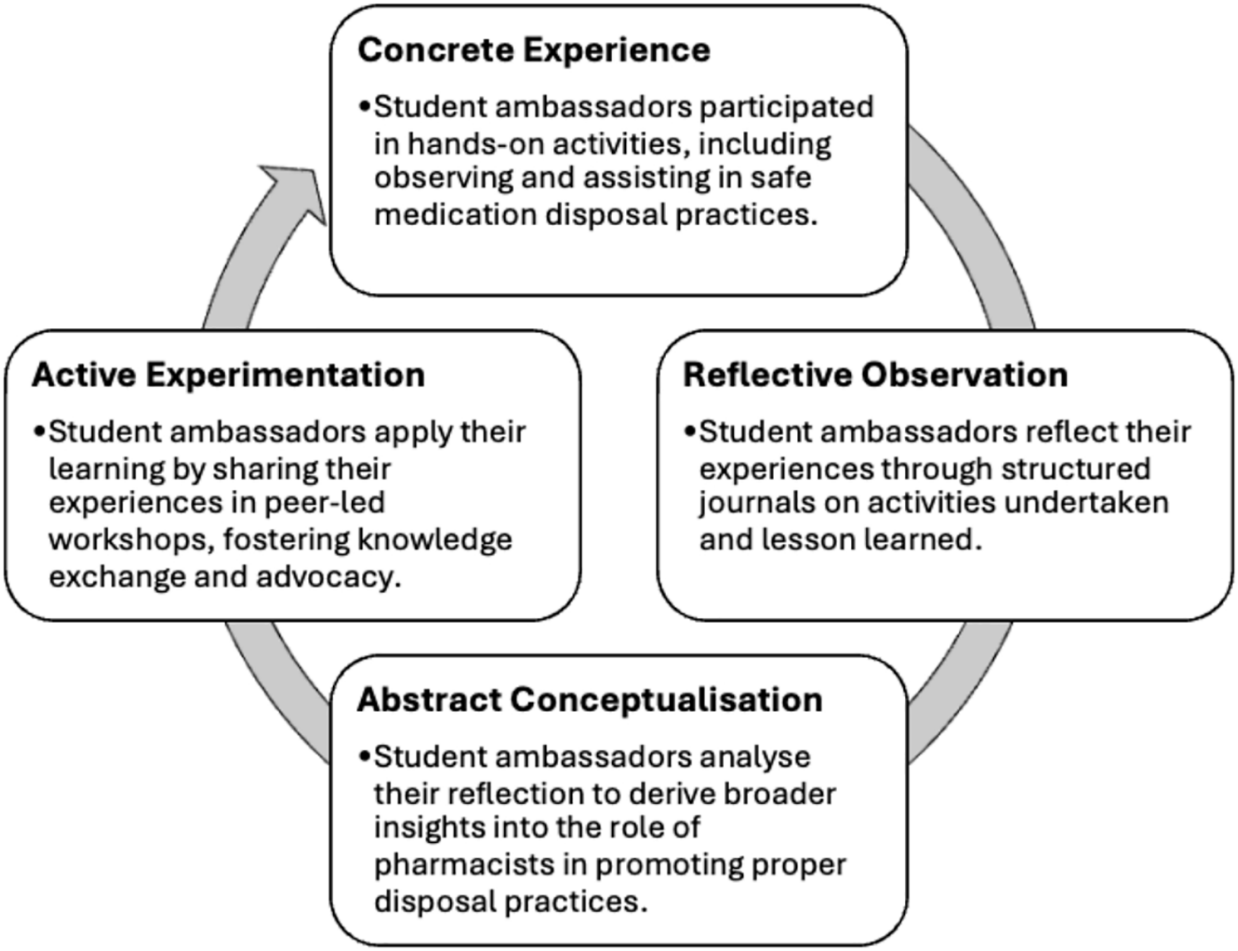
Adapted Kolb’s Experiential Learning Cycle (ELC) applied to Phase 1 safe medication disposal training. The model illustrates how student ambassadors progressed through Concrete Experience (hands-on participation), Reflective Observation (structured journaling), Abstract Conceptualisation (deriving insights), and Active Experimentation (peer-led workshops).

According to Kolb, learning occurs through the interaction of theory and experience [15]. His ELC conceptualises learning as a dynamic process consisting of four interconnected stages: Concrete Experience, Reflective Observation, Abstract Conceptualisation, and Active Experimentation (Fig 1). Engaging in all four stages foster a deeper understanding and skill development, ensuring a comprehensive learning experience.

In Phase 1 of this study, Kolb’s ELC was embedded to engage student ambassadors in an iterative learning process. Students first gained direct exposure to safe medication disposal practices through hands-on activities (Concrete Experience). This was followed by reflecting their learning experience (Reflective Observation) and developing conceptual understanding based on their insight (Abstract Conceptualization). Finally, they applied their knowledge in practice by leading peer-led workshops, promoting knowledge exchange and advocacy (Active Experimentation). Through this cycle, student ambassadors not only reinforced their own learning but also inspired more pharmacy students to join the initiative. As new ambassadors engage in experiential learning, they sustain the learning cycle and expand the reach of the intervention. This approach deepened students’ comprehension of safe medication disposal while fostering long-term engagement and leadership development within pharmacy education.

**Fig 2:**
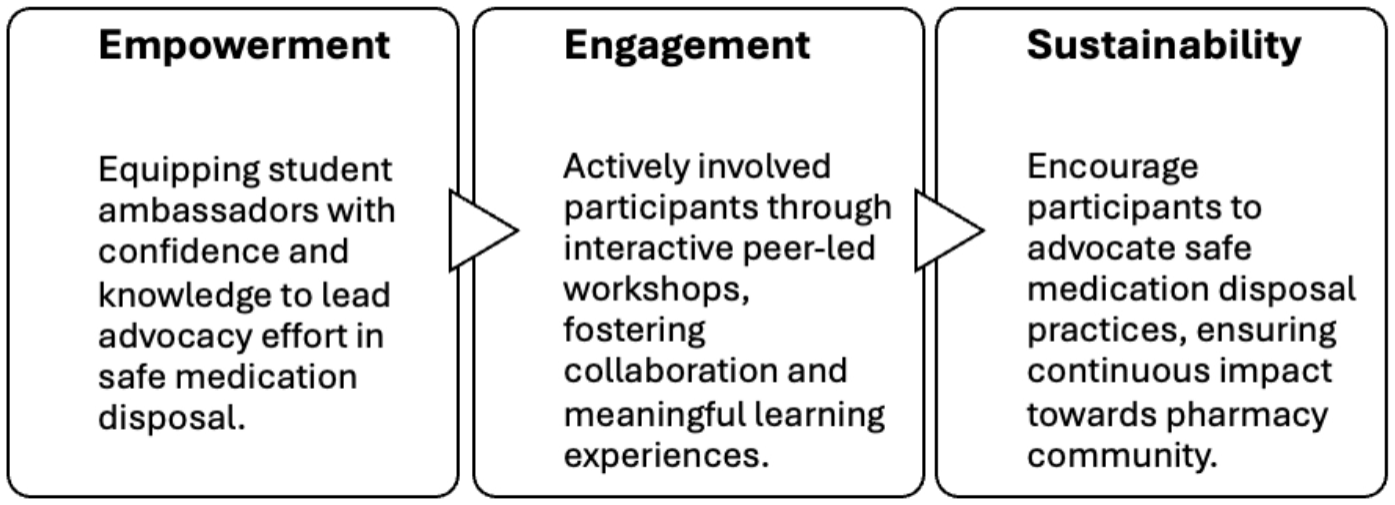
Adapted Peer Education Framework applied in Phase 2 of the study. The framework highlights three dimensions of peer-led learning: Empowerment (building advocacy confidence), Engagement (interactive workshops fostering collaboration), and Sustainability (encouraging continuous advocacy in the pharmacy community).

Building on this foundation, the Peer Education Framework (Fig 2) was used as an implementation guide for Phase 2, focusing on how student ambassadors transitioned from learners to peer educators after completing the final “active experimentation” stage of Kolb’s ELC (Fig 1). Instead of reiterating theoretical components, the framework informed the design of workshop activities; such as student-led discussions, scenario-based teaching, and collaborative knowledge sharing; and supported the interpretation of Phase 2 findings by highlighting how empowerment, peer influence, and engagement mechanisms contributed to shifts in knowledge and advocacy motivation [16].

By training student ambassadors as peer educators, the study created a cascading effect, broadening the reach of safe medication disposal education and fostering long-term behaviour change. Together, Kolb’s ELC and the Peer Education Framework provided a structured, student-centred intervention that bridged educational gaps in pharmacy training, promoted active and peer-driven learning, and supported the sustainability of safe medication disposal practices within the healthcare community.

Kolb’s ELC guided the structure of Phase 1 by determining how student ambassadors progressed through hands-on engagement, structured reflection, conceptual synthesis and subsequent application in the peer-led workshop. Rather than restating the full theory, the model was used to sequence learning activities, ensuring that reflective journals captured observation and meaning-making, while the design of Phase 2 represented the final “active experimentation” stage. The Peer Education Framework, in turn, shaped Phase 2 by outlining mechanisms for student-to-student influence, informing decisions about workshop facilitation, empowerment strategies, and the interpretation of how peer-led teaching contributed to shifts in knowledge and advocacy readiness [15,16].

Together, the two frameworks shaped not only the design of each study phase but also the analytic interpretation by clarifying how experiential reflection and peer influence contributed to observed cognitive and motivational shifts.

### Methodology

#### Study design and settings.

**Fig 3:**
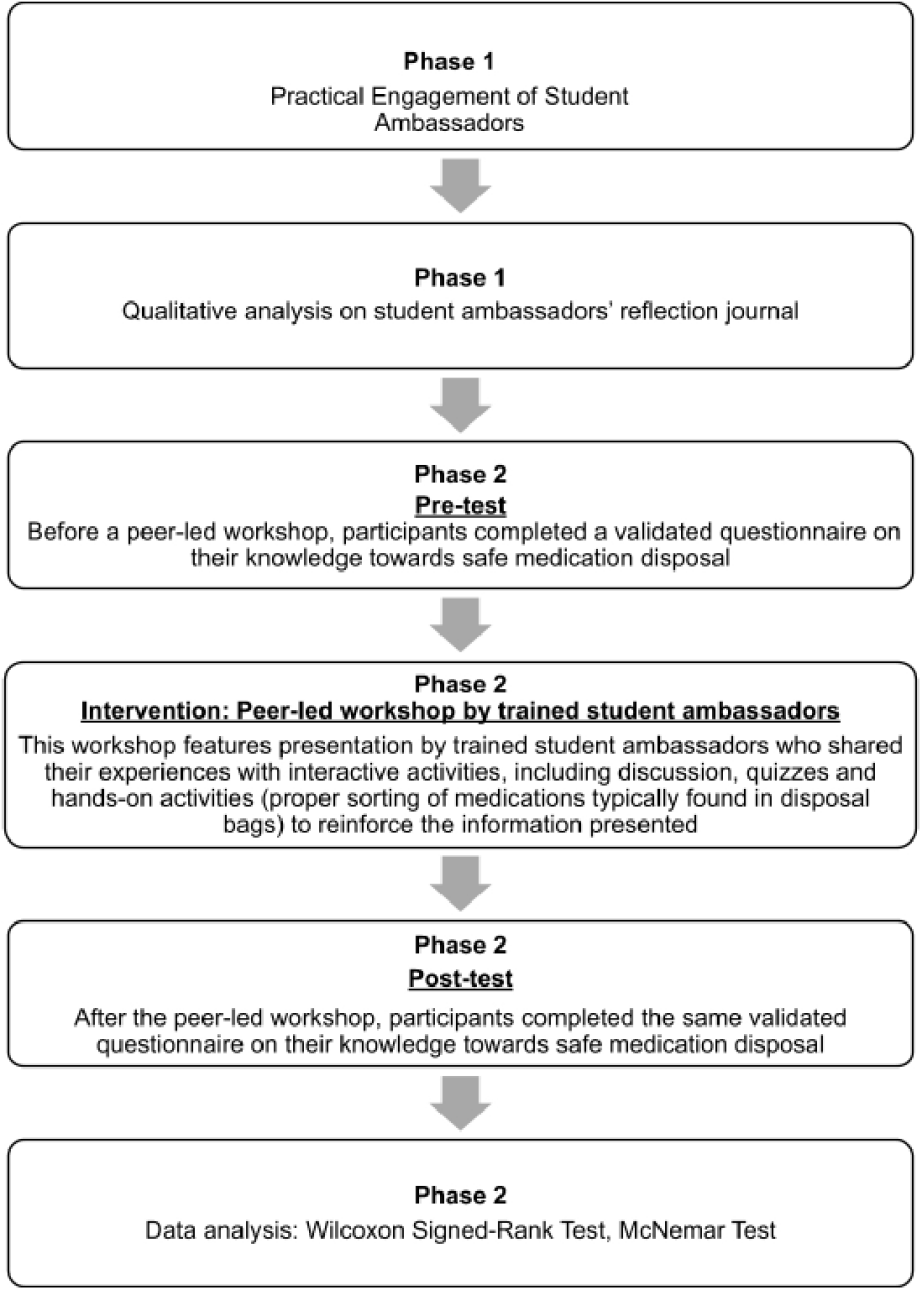
Flow of the study design. The study was conducted in two phases. Phase 1 involved the practical engagement of student ambassadors in safe medication disposal activities, followed by qualitative analysis of their reflection journals. Phase 2 consisted of a peer-led educational intervention with undergraduate pharmacy students. Participants first completed a validated pre-test questionnaire, then attended a peer-led workshop conducted by trained student ambassadors, and finally completed the same post-test questionnaire. Knowledge improvement was evaluated using the Wilcoxon Signed-Rank Test, while item-level changes were assessed with the McNemar Test.

We employed a mixed-method educational research design conducted over two phases. In Phase 1, qualitative data was collected to explore student ambassadors’ experiences and perceptions with their participation in safe medication disposal programs organised by community pharmacies. In Phase 2, quantitative data was gathered to assess participants’ knowledge towards safe medication disposal before and after a student ambassadors-led workshop. The study flow is illustrated in Fig 3.

#### Phase 1: Practical engagement of student ambassadors.

Selected student ambassadors participated in a structured practical engagement program conducted at community pharmacy settings in March 2024 (1/3/2024–28/3/2024). Student ambassadors were selected based on voluntary interest in community-based pharmacy activities, which may introduce self-selection bias, as students who chose to participate were likely already motivated or predisposed toward public health and advocacy-related initiatives. To ensure consistency in preparation, all student ambassadors received the same structured training delivered by pharmacists from the professional network, following a standardised set of instructions and expectations; however, individual differences in engagement and reflective depth may have contributed to variability in learning experiences.

During this phase, student ambassadors engaged in hands-on activities related to safe medication disposal, including observing and assisting with medication return and disposal process. These activities aimed to build their practical knowledge and advocacy skills. Each ambassador documented their experiences in a structured reflective journal, detailing the activities undertaken, lessons learned, challenges encountered, and successes achieved. The reflective journal prompts were developed specifically for this study to align with Kolb’s Experiential Learning Cycle. Four guiding questions were created to encourage students to articulate (1) concrete observations during engagement, (2) reflections on what they learned, (3) conceptual insights linking their experience to professional roles, and (4) anticipated future actions related to advocacy or public education. These prompts were reviewed by three researchers with experience in qualitative pedagogy to ensure clarity, relevance, and alignment with experiential learning theory.

The reflections were compiled and analysed qualitatively to identify recurring themes and insights into the ambassadors’ learning experiences. The findings from this phase informed the development of Phase 2 peer-led workshop, ensuring that real-world experiences translated into meaningful peer education. Reporting of the qualitative phase adheres to the consolidated criteria for qualitative research (COREQ) to ensure methodological transparency and rigor.

#### Phase 2: Peer-led workshop.

Building on their practical engagement, trained student ambassadors facilitated a peer-led safe medication disposal workshop in May 2024 (24/5/2024). This workshop aimed to educate and inspire undergraduate pharmacy students to integrate safe medication disposal practices into their future professional roles.

Participants for this workshop were recruited through convenience sampling via email invitations and social media announcements shared by pharmacy student representatives from public and private universities across Malaysia. In total, 43 students provided informed consent and attended the workshop. As recruitment occurred through convenience sampling, participants may differ systematically from non-respondents, introducing additional self-selection bias. Such bias may have resulted in higher baseline interest or familiarity with pharmaceutical waste topics among those who chose to participate. Pharmacy undergraduate students from Year 1 to Year 3 in 2024 were invited to participate. Those who were unable to attend the workshop and unable to understand English were excluded from the study.

During the peer-led workshop, trained student ambassadors shared their training experiences and practical engagement while educating participants on proper medication disposal methods. This session provided insights into their journey and fostered an interactive learning environment through discussion and quizzes that reinforced key concepts. Pre- and post-tests were conducted to assess participants’ knowledge level towards safe medication disposal.

Insights from Phase 1 played a direct role in shaping the content and structure of the Phase 2 peer-led workshop. Thematic analysis of student reflections revealed recurring gaps in knowledge (e.g., uncertainty regarding environmentally appropriate disposal methods and the scope of pharmacist responsibility), as well as challenges such as lack of public awareness and limited access to disposal facilities. These themes guided the selection of workshop topics, which emphasized areas where students demonstrated limited confidence. Additionally, student-reported challenges; such as communicating risks to the public and clarifying misconceptions, were incorporated into case scenarios, discussion prompts, and quiz items. This process reflects the transition from Reflective Observation to Active Experimentation in Kolb’s ELC, where insights gained through experience informed the design of a targeted educational intervention.

#### Pre- and post-intervention assessments.

To assess the effectiveness of the peer-led workshop, the one-group pretest post-test experimental design was employed, as proposed by Fraenkel and Wallen [17]. Prior to the workshop, participants completed a validated questionnaire assessing their knowledge of safe medication disposal. The same questionnaire was administered after the workshop to measure knowledge improvement.

#### Instrument.

Based on the topic covered during the peer-led workshop, a validated questionnaire of 10 questions was given to the participants [18,19]. The questionnaire was adapted from a locally developed and validated instrument assessing knowledge related to safe medication return and disposal (ReDiUM) [18]. In the validation study, the 10-item knowledge domain demonstrated acceptable reliability (Cronbach’s α ≈ 0.70) and 2-week test–retest agreement (Cohen’s κ: fair–moderate), supporting its use in the local context [18]. The questionnaire was in English and distributed through Google Forms. The same set of questionnaires was used for the pre-test and post-test.

Answers to the pre-test and post-test were in the form of either “Yes”, “No”, or “Not Sure”. A maximum score of 10 was possible for each test when all the questions were answered correctly. Correct answers were given one mark, while incorrect answers were marked with zero.

#### Data analysis.

In Phase 1, open-ended questions from students’ reflective journals were compiled and analysed qualitatively to identify recurring themes. The analysis focused on the ambassadors’ learning experiences, the effectiveness of practical engagement, and how these experiences influence their ability to advocate for safe disposal. Two researchers (YLL, SPS) independently coded 5 reflections to develop a set of initial themes and met up to discuss and research a consensus on the codes and coding rules. The coding process followed an iterative, multi-step approach. First, an initial codebook was developed inductively from an exploratory reading of five reflection journals by two researchers. The researchers compared codes and refined the codebook through discussion to ensure conceptual clarity and eliminate redundancies. Interrater reliability was assessed using percentage agreement, with >80% agreement considered indicative of good reliability for exploratory thematic analysis. Discrepancies were resolved through consensus meetings involving all three researchers (YLL, SPS, LJJ), during which code definitions were revised and exemplar quotes were re-examined until agreement was reached. Following finalisation of the codebook, the two coders independently applied the agreed-upon codes to the full dataset. Themes were interpreted deductively using Kolb’s ELC to map students’ experiential progression across stages of learning [15].

In Phase 2, data were analysed using the Statistical Package for Social Sciences (SPSS), version 29. Descriptive analysis was performed to describe the sample distribution and demographic characteristics. Pre-test and post-test data were statistically analysed to assess changes in participants’ knowledge. Knowledge levels were categorized into three tiers based on Bloom’s cutoff criteria: high (80–100%), moderate (60–79%), and low (<60%) [20]. For a maximum score of 10, the corresponding ranges were as follows: high (8–10; 80–100%), moderate (6–7; 60–79%), and low (0–5; < 60%). The Wilcoxon signed-rank test was used to compare pre- and post-intervention scores within the same group, while the McNemar test was applied for categorical data analysis. Effect size (r) was calculated for Wilcoxon tests using formula $r\;=\;Z\;/\;\sqrt{N}$, where *Z* is the standardized test statistic and *N* is the number of observations [21]. Cohen’s criteria (0.1 = small, 0.3 = medium, 0.5 = large) were applied for interpretation [22]. A significance level of p < 0.05 was considered statistically significant. Interpretation of these results was guided by the Peer Education Framework, which informed understanding of how the workshop fostered empowerment, engagement, sustainability and improvements in knowledge among participants.

### Ethical consideration

Ethical approval for the study was obtained from the Monash University Human Research Ethics Committee (MUHREC) (Project ID: 40604). Informed consents were obtained from the participants prior to the administration of the survey. They were provided with an explanatory statement and were assured confidentiality of their data.

## Result

### Phase 1

A total of 35 undergraduate pharmacy students participated as student ambassadors, all of whom completed Phase 1 of the study without attrition. The qualitative analyses of the student ambassadors’ reflective diaries generated five themes on their experiences and reflections on safe medication disposal practices. These themes offered insights into their awareness, attitudes, and motivations, as well as the challenges they perceive in promoting safe medication disposal practices. Table 1 presented the coding structure that illustrated each theme.

**Table 1:** Coding structure for each theme derived from student ambassadors’ reflection journals (Phase 1). Thematic analysis identified five key themes related to safe medication disposal: awareness and education, environmental and health risks of improper disposal, personal responsibility and advocacy, challenges in promoting safe medication disposal, and engagement and motivation for continuous improvement. Each theme was supported by sub-codes that captured specific dimensions of students’ experiences and perceptions.

Theme	Codes
Awareness and Education on Safe Medication Disposal	Raising awareness Community engagement Promoting proper disposal Organizing educational events
Environmental and Health Risks of Improper Disposal	Environmental contamination Health risks from improper disposal Dangers of flushing drugs Protecting ecosystems
Personal Responsibility and Advocacy	Taking responsibility Advocating for safe disposal Encouraging proper practices Driving community change
Challenges in Promoting Safe Medication Disposal	Bridging knowledge gaps Shifting public behavior Addressing disposal challenges Improving disposal infrastructure
Engagement and Motivation for Continuous Improvement	Taking action and seeking involvement Committed to improvement Exploring better practices Promoting sustainable healthcare practices

#### Theme 1: Awareness and education on safe medication disposal.

Student ambassadors reflected on their initial lack of awareness regarding proper disposal methods before participating in engagement programs. Beyond individual reflections, the themes illustrated a developmental shift in how pharmacy students conceptualised their professional role. Students moved from perceiving medication disposal as a technical task to recognising its broader environmental and public health implications. Their reflections suggested an emerging professional identity in which advocacy becomes integral rather than peripheral. This pattern reflected the reflective–conceptual stages of Kolb’s cycle, where learners transformed isolated experiences into integrated understanding that shapes future practice behaviours. One student noted that safe medication disposal was not explicitly covered in their pharmacy curriculum, while another admitted that his prior understanding was limited to concerns about consuming expired medications rather than proper disposal methods:

*“Indeed, prior to attending this (out-of-class) event on safe medication disposal (in the community pharmacy), I was unaware of the proper methods for disposing of medications. Typically, I would simply discard them in the regular trash bin.” (S2)*

*“This engagement program enabled me to learn beyond the university curriculum, which is useful for my future career.” (S31)*

*“Prior to this visit, I was only aware of the danger of consuming expired medication.” (S34)*

Through experiential learning, students gained valuable knowledge into the environmental and public health risks of improper medication disposal. They recognized the importance of pharmacist-led education in promoting safe disposal practices. Following the engagement, students expressed a strong commitment to educating their communities. They proposed leveraging outreach platforms, such as social media and public campaigns, to enhance public awareness.

*“Raising public awareness on social media by addressing the importance of safe medication disposal and the negative consequences of disposing medication wrongly.” (S11, S29)*

*“As university students, we can take the lead by organizing campaigns and engaging with the public to make proper disposal a well-known practice.” (S4, S7, S8)*

#### Theme 2: Environmental and health risks of improper disposal.

Student ambassadors highlighted the serious consequences of improper medication disposal, including water contamination, antibiotic resistance and ecological harm. They recognized its impact on both aquatic life and public health, with student noting,

*“Flushing or discarding medications improperly can introduce harmful substances into our water systems, affecting both marine ecosystems and human health.” (S6)*

*“Improper disposal of antibiotics can contribute to the development of antibiotic resistance, leading to the emergence of “superbugs” that are difficult to treat and pose serious threats to human life.” (S3)*

Beyond environmental concerns, students also recognized the risks of accidental drug ingestion, particularly for vulnerable groups such as children and the elderly. Students pointed out,

*“Improper disposal not only harms the environment but also increases the risk of unintentional drug consumption, which can lead to poisoning or medication misuse in households.” (S8)*

*“Improper disposal of medication, for example, throwing the pills into the bin may lead to children accidentally ingesting them as the pills are often colourful and may be mistaken as candy.” (S12)*

#### Theme 3: Personal responsibility and advocacy.

Many students reflected on their personal responsibility as future pharmacists to advocate for safe medication disposal. Despite individual differences in how students expressed their reflections, a consistent pattern emerged: experiential engagement strengthened their awareness of the pharmacist’s role in public health advocacy. Rather than simply acquiring knowledge, students described a broader shift in professional identity, recognising that counselling on medication disposal contributes to environmental stewardship and community well-being. This sense of responsibility was accompanied by a willingness to engage in future outreach and integrate disposal education into routine patient interactions. One student shared:

*“This visit is an eye-opening experience for me. I can make a difference in the future by starting to educate the people around me on the importance of safe medication disposal.” (S35)*

Students emphasized their dedication to advocacy:

*“(I am) committed to spread this awareness to the people around me including my family, relatives, and friends. If possible, as a pharmacy student, I am also responsible to teach the public by organizing activities at the campus or in public to spread awareness.” (S8)*

*“This visit made me realize the importance of safe medication disposal and inspired me to take responsibility as a future pharmacist and citizen by practicing proper disposal and educating my friends and family to do the same.” (S11)*

#### Theme 4: Challenges in promoting safe medication disposal.

While students recognized the importance of safe medication disposal, they also acknowledged significant challenges in raising awareness and changing public behaviours. They identified gaps in public knowledge, inconsistencies in disposal practices, and a lack of accessible disposal infrastructure as key barriers. One student remarked,

*“Many people still don’t realize the consequences of improper medication disposal. There’s a long way to go in ensuring that safe disposal becomes a common practice.” (S10)*

Students also highlighted convenience as a significant obstacle:

*“It might be inconvenient for the community if they are far from a safe medication disposal service provider, which discourages proper disposal.” (S25)*

*“Convenience plays a huge role—many patients are unwilling to travel to a pharmacy or hospital just to dispose of their medications properly.” (S34)*

Beyond public awareness and convenience, students also encountered practical difficulties in handling and sorting medication waste. One participant reflected on the challenges of identifying medication due to missing labels:

*“One of the challenges I faced in categorizing the medications was the absence of indication labels. All the medications were mixed together, making it difficult to determine their intended use.” (S2)*

#### Theme 5: Engagement and motivation for continuous improvement.

Students expressed strong motivation to engage further with safe medication disposal initiatives. Many showed interest in organizing future site visits, workshops, and awareness campaigns. While others suggested enhancing hands-on activities by exploring medication disposal logistics in more depth. One student highlighted the value of hands-on learning in motivating them for future advocacy:

*“This hands-on learning experience is invaluable. It provides concrete examples of how pharmacists can contribute to public health and environmental safety beyond the pharmacy counter. It has motivated me to advocate for proper medication disposal and educate others.”(S22)*

Another student shared how observing medication disposal in practice deepened their understanding of waste management:

*“I had the opportunity to observe firsthand the volume and types of medications being disposed of daily. This experience provided a practical perspective that complimented my previous theoretical knowledge of medication waste management.” (S3)*

Additionally, a student expressed interest in gaining more comprehensive understanding of the disposal process:

*“I’d love to see the entire journey of disposed medications—from collection to final disposal—to fully understand its impact.” (S1)*

Overall, the experiential learning fostered a sense of responsibility and advocacy among students, equipping them with practical knowledge and inspiring them to contribute to improved medication disposal practices in their future careers.

More specifically, the themes showed that experiential engagement fostered both cognitive and affective shifts. Students not only gained technical understanding but also articulated growing confidence, ethical responsibility, and a sense of agency as future pharmacists. These deeper interpretive patterns extend beyond descriptive accounts and highlight the mechanism by which experiential learning triggers internalisation of advocacy roles—an essential intention of the intervention design.

Importantly, these insights indicated that experiential learning was most effective when it linked practical exposure with structured reflection and targeted skill development, rather than relying on experience alone. The ambassadors’ reflections demonstrated progressive movement through Kolb’s learning stages, but also revealed the limits of unstructured exposure; highlighting the need for clearer instructional scaffolding and opportunities for iterative feedback. This consolidated interpretation helped explain why experiential learning contributed meaningfully to motivation and advocacy, while also helped identify boundaries of its effectiveness in shaping more specialised knowledge domains.

## Phase 2

A total of 43 undergraduate pharmacy students participated in the peer-led workshop conducted by trained student ambassadors. Most of the participants were female, 76.7% (n = 33), with a median age of 22, as depicted in Table 2.

**Table 2:** Demographic characteristics of undergraduate pharmacy students who participated in the peer-led intervention (n = 43). The majority of participants were in their second year of study (55.8%) and most were female (76.7%).

Characteristics	No of participants (n, %)
**Year of study**	
Year 1	3 (7.0%)
Year 2	24 (55.8%)
Year 3	16 (37.2%)
**Gender**	
Male	10 (23.3%)
Female	33 (76.7%)

**Fig 4:**
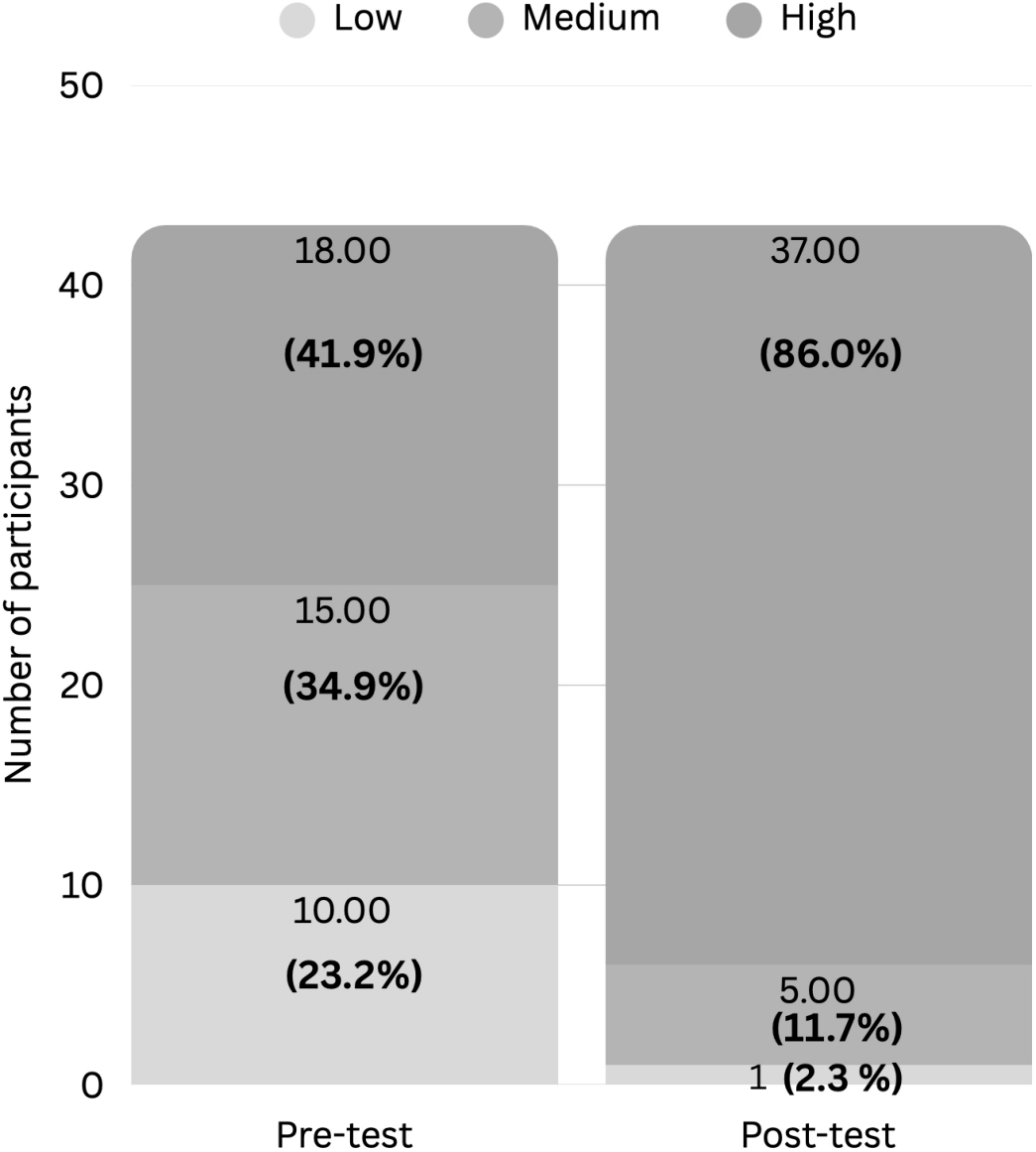
Distribution of knowledge levels on safe medication disposal before and after the peer-led workshop (n = 43). The proportion of students with high knowledge increased substantially from 41.9% at pre-test to 86.0% at post-test, while the proportion with low knowledge decreased from 23.2% to 2.3%. These findings highlight the impact of peer-led education in shifting students from lower to higher knowledge categories.

**Fig 5:**
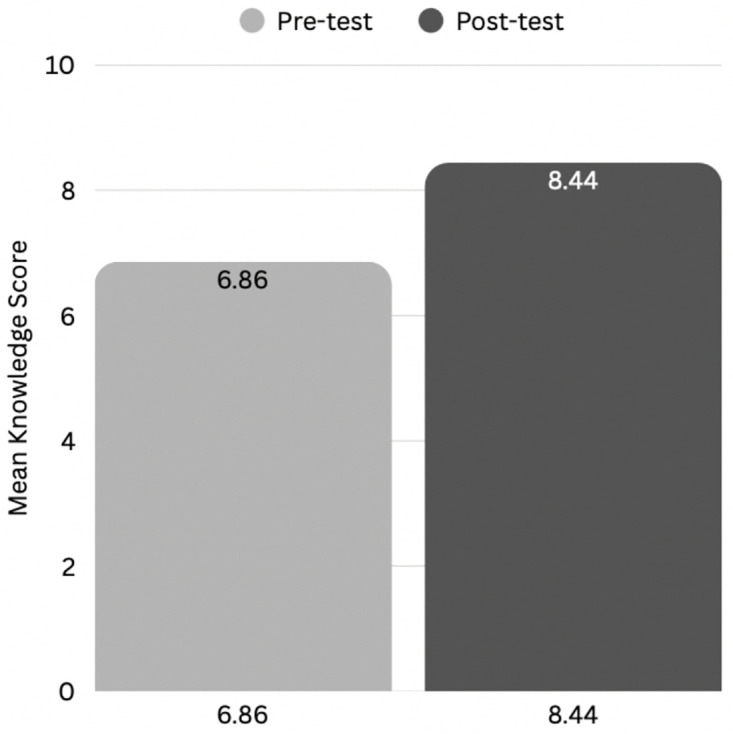
Effect of intervention on the knowledge level of safe medication disposal among undergraduate pharmacy students (n = 43). Mean knowledge scores increased significantly from pre-test (6.86) to post-test (8.44) following the peer-led workshop, demonstrating the effectiveness of the intervention.

Participants’ knowledge levels before and after the peer-led workshop intervention were presented in Fig 4. Pre-test knowledge levels were as follows: 23.2% (n = 10) low, 34.9% (n = 15) moderate, and 41.9% (n = 18) high. Post-test only 2.3% (n = 1) had low knowledge, while 11.7% (n = 5) had moderate knowledge, and 86% (n = 37) were classified as having high knowledge. These findings highlighted the impact of peer-led education in shifting students from lower to higher knowledge categories. Overall, participants’ mean knowledge score of safe medication disposal significantly increased from 6.86 to 8.44, indicating a meaningful enhancement in understanding proper medication disposal (Fig 5). The Wilcoxon signed-rank test (Z = −4.827; p < 0.001) confirmed a statistically significant improvement in knowledge (Table 3). The corresponding effect size was large (r = 0.74) according to Cohen’s benchmarks, indicating a substantial impact of peer-led workshops on participants’ knowledge (Table 3) [22]. Table 4 presented a summary of the results for each question, comparing pre-test and post-test assessments. In the pre-test, more than 75% of participants answered items 1, 3, 4, 5, 7 and 8 correctly. However, knowledge was poor in items 2, 6, and 9, with less than 50% correct answers, and moderate in item 10, with a correct score in 50% − 75%. Post-test results demonstrated significant improvements in all items with initially low pre-test scores.

**Table 3:** Effect of the peer-led intervention on mean knowledge scores of safe medication disposal among undergraduate pharmacy students (n = 43). The mean knowledge score improved from 6.861 to 8.442 after the peer-led intervention (mean difference = 1.581, + 23.0%). The Wilcoxon Signed-Rank Test confirmed a significant improvement (Z = –4.827, p < 0.001), with a large effect size (r = 0.74).

Knowledge level (n = 43)						
Mean score		Mean difference (Post – Pre)	% of change from baseline	Z	p-value^a^	Effect size (r)ᵇ
Pre-test	Post-test					
6.861	8.442	1.581	+ 23.0%	− 4.827^a^	<0.001*	0.74

^a^ Wilcoxon Signed Ranks Test, *statistical significance at p < 0.05.

ᵇ Effect size (r) was calculated using the formula r = Z/ √N$\text{r}\;=\;\text{Z}/\sqrt{N}\$<delclass="ice-delice-cts-1"data-cid="13"data-userid="1"data-rolename="Author"data-username="a.q.j.blebil@swansea.ac.uk,aliblebil@yahoo.com"data-changedata=""data-time="1776093926309"data-last-change-time="1776093926308"data-action-time="1776346169899"data-action-user="collation@nkw.pub"data-action="Accepted"data-action-old-tag="del"data-action-hidden="Yes">\$</del>$ Effect size interpretation follows Cohen’s criteria (0.1 = small, 0.3 = medium, 0.5 = large).

**Table 4:** Knowledge of participants on safe medication disposal before and after the peer-led intervention (n = 43). Item-level analysis showed significant improvements in several areas. Significant improvements were observed for wastewater treatment inefficacy, incineration benefits, disposal of solid medicines and cream/ointments (p < 0.05).

Question		Correct answer		
		Pre-test (Mean %)	Post-test (Mean %)	p-value^b^
**1.**	Improper drug disposal has harmful effects on the environment and ecosystem.	95.35	97.67	1.000
**2.**	Wastewater treatment removes most of the medicines from the environment and ecosystem.	48.84	83.72	<0.001*
**3.**	It is acceptable to dispose of solid medicines (such as tablets, capsules and patches) in the garbage.	76.74	97.67	0.004*
**4.**	It is acceptable to dispose of liquid medicines by throwing down the sink.	93.02	97.67	0.500
**5.**	It is acceptable to dispose of medicines by flushing down the toilet.	93.02	97.67	0.500
**6.**	Incineration is the environmentally sound way of disposing of unwanted medicines.	20.93	62.79	<0.001*
**7.**	It is acceptable to dispose of needles and syringes in the garbage.	93.02	95.35	1.000
**8.**	It is acceptable to return or dispose of unused medicines to a local pharmacy or healthcare facility.	88.37	95.35	0.453
**9.**	It is acceptable to dispose of pressurized aerosol metered-dose inhalers (like the Ventolin inhaler) in the garbage.	11.63	25.58	0.146
**10.**	It is acceptable to dispose of creams and ointments in the garbage.	65.12	90.70	0.007*

^b^McNemar Test, *statistical significant at p < 0.05.

## Discussion

### Experiential learning and the role of pharmacy students in medication disposal advocacy

Our study highlights the urgent need to increase awareness and education on safe medication disposal among pharmacy students, preparing them to serve as key advocates in promoting proper disposal practice. In this study, pharmacy students recognized their responsibility in educating others and disseminating knowledge on proper disposal practices, aligning with previous research indicating that healthcare professionals play a pivotal role in influencing patient behaviours [23]. While public health education strategies such as social media, university campaigns, and workshops have been widely advocated to raise awareness, pharmacy students can serve as key influencers in promoting safer medication disposal [2,6,24]. Internationally, similar patterns have been observed. Urslak et al. reported that pharmacy schools across multiple countries have increasingly incorporated planetary health content, emphasising how pharmaceutical waste impacts the environment and mitigation strategies [25]. Research from Canada indicates that pharmacy education has yet to establish unified standards for environmental sustainability competencies, with initiatives to date largely fragmented and limited to individual courses [26]. Global organisations such as the International Pharmaceutical Federation have also highlighted improper medicine disposal as both a public health and environmental risk, underscoring the need to prepare students as future advocates in this area [27,28]. Embedding safe medication disposal more systematically into curricula will strengthen students’ ability to contribute effectively to both public health and environmental stewardship, aligning with United Nations Sustainable Development Goals (SDGs) [28]. These international findings contextualise our results and highlight how peer-led experiential models can complement emerging global priorities in pharmacy education, while also revealing system-level gaps that local adaptations must address.

Building on these global trends, it is important to consider how local structural and curricular factors shape the applicability and scalability of such educational models. In Malaysia, the transferability of these findings may be influenced by differences in disposal infrastructure, curriculum expectations and sociocultural norms. Disposal infrastructure varies across regions, with limited availability of formal take-back programs outside urban areas, which may magnify the challenges students observed in practice [29]. In addition, the Pharmacy Board of Malaysia accreditation standards specify core bodies of knowledge for undergraduate pharmacy programmes while allowing institutional autonomy in curriculum design. Environmental health or ecopharmacology-related content is not explicitly listed as a core topic and therefore may be covered with varying depth across institutions [30]. Cultural norms regarding waste management and household medicine storage may further influence how the public responds to pharmacist-led counselling [29]. These contextual nuances suggest that while the educational model shows promise, its implementation and impact may differ in countries with more established pharmaceutical waste systems or differing curricular standards.

Our study also revealed strong student motivation to engage further in safe medication disposal initiatives. Our findings demonstrate that experiential learning through direct engagement in medication disposal practices significantly enhances students’ understanding of regulatory challenges and public misconceptions. In Phase 1 of this study, student ambassadors were exposed to site visits and interactive learning opportunities to deepen their understanding of disposal systems. According to Kolb’s Experiential Learning Theory, learning is most effective when individuals actively engage in experiences, reflect on them, and integrate insights into future practice [15]. Active participation fosters intrinsic motivation, critical thinking and deeper engagement, making learning more meaningful than passive learning methods such as lectures [31,32]. Experiential learning has also been emphasized globally as an essential component in the education of future pharmacists, facilitating the transition from theoretical knowledge to applied practice. Consistent with our findings, experiential learning in the United States and Canada has been shown to build professionalism, enhance communication confidence, and strengthen understanding of multidisciplinary team collaboration [33,34]. These findings highlight the value of experiential learning as a globally adaptable educational model. Additionally, experiential learning encourages students to analyse situations critically, make informed decisions, and develop problem-solving skills, which are essential for professional success [15]. Integrating structured experiential learning into the pharmacy curriculum will better prepare students as future advocates for safe medication disposal, reinforcing their role in environmental sustainability and patient safety.

The integration of findings across phases highlights how experiential reflection informed subsequent peer-led teaching. Qualitative themes from Phase 1 not only revealed students’ growing awareness and advocacy motivation but also identified specific areas of uncertainty, particularly regarding environmental risks, safe disposal pathways, and the logistical challenges of take-back programs. These insights shaped the workshop content in Phase 2, ensuring alignment between learners’ identified needs and the educational focus of the intervention. This analytical connection demonstrates the iterative nature of the study design and reflects both Kolb’s learning cycle. where reflection informs future action and the Peer Education Framework, which emphasizes empowerment followed by structured engagement.

A strong sense of personal responsibility and advocacy emerged among students, reflecting their recognition of the pharmacist’s role in promoting safe medication disposal. Previous studies have highlighted the crucial role of healthcare professionals, including prescribers and pharmacists, in educating communities on proper medication disposal methods [35]. In Taiwan, pharmacy students’ attitudes towards professionalism with designed experiential learning programme in the community setting have fostered positive attitudes towards altruism, accountability and duty [36]. In our study, many students expressed their intention to educate their families and communities, emphasizing the importance of incorporating responsible disposal practices into daily life. This aligns with research suggesting that education and advocacy efforts by pharmacists and pharmacy students can enhance public awareness and encourage proper medication disposal practices [2]. By fostering a culture of advocacy, pharmacy students can drive long-term improvements in medication disposal awareness and practices.

Despite its benefits, experiential learning presents challenges, including difficulties in assessment due to the subjective nature of learning, variability in student engagement and logistical constraints due to site capacity [37,38]. Without proper structure and reflection, students may struggle to grasp essential concepts, reducing the effectiveness of such programs. To address these challenges and maximize the benefits of experiential learning, educators should collaborate with community pharmacies to co-design structured engagement programs, including mentorship and practical training in safe medication disposal.

Students also acknowledged various challenges in promoting safe medication disposal. Public behaviour change is often hindered by knowledge gaps, limited access to disposal facilities, and lack of standardized disposal policies, all of which have been highlighted as barriers in previous studies. Akande-Sholabi et al. identified significant barriers, including lack of access to medication take-back programs (50.4%), lack of knowledge (36.8%) and inconveniences (10.3%) [10]. Some students noted that many patients are unwilling to travel to pharmacies or hospitals solely for medication disposal purposes, highlighting a key barrier to medication take-back programs. To address this, expanding medication take-back facilities to private healthcare settings, including community pharmacies, private clinics and hospitals could improve accessibility and encourage greater public participation and safe medication disposal [39]. Overcoming these challenges requires a multifaceted approach, including policy interventions, improved infrastructure, and pharmacist-led educational campaigns tailored to different community needs.

### Effectiveness of peer-led workshop in educational intervention

In Phase 2, our findings demonstrate a significant improvement in participants’ knowledge post-intervention, particularly in understanding that wastewater treatment is ineffective at removing most pharmaceuticals from the environment, incineration is the most environmentally sound method for disposing of unwanted medications, and the correct way to discard solid medicines and metered dose inhaler (MDIs). Participants also expressed significant concerns about the environmental and health risks associated with improper medication disposal, reinforcing existing research on pharmaceutical pollution, which has shown that flushing medications down the drain contributes to water contamination and the emergence of antibiotic resistance [40–42].

Overall, participants’ knowledge regarding the safe disposal of unused medications increased significantly after the peer-led workshop (mean score 6.86 to 8.44 out of 10, p < 0.001), indicating a substantial improvement following the intervention. In practical terms, these improvements suggest that many students shifted from moderate to higher knowledge level, which is supported by the observed increase in the proportion classified as “high knowledge” post-intervention from n = 18 (41.9%) pre-intervention to n = 37 (86.0%) post intervention. However, improvements were not uniform across domains. Item-level results indicated that specialised disposal topics, particularly handling procedures for pressurised metered-dose inhalers, remained persistently low despite the workshop, suggesting that brief peer-led sessions may not provide sufficient depth for more technical topics and targeted emphasis on specialised waste-handling practices is needed. Student ambassadors also reported varied confidence levels when facilitating discussions, indicating that additional training or rehearsal time may strengthen delivery consistency. Future iterations could incorporate demonstration videos, structured case simulations, or extended practice time to enhance comprehension of complex disposal scenarios and improve facilitator consistency.

Although this study did not assess behavioural outcomes longitudinally, qualitative reflections illustrate that many students linked increased knowledge with an intention to counsel others, adopt proper disposal practices personally, and advocate for environmentally responsible medication handling. This alignment between knowledge gains and stated willingness to act suggests early internalisation of advocacy behaviours, consistent with experiential and peer-led learning models. These findings are consistent with prior evidence that educational initiatives can improve medication disposal knowledge and awareness. Lai et al. reported significant improvements in public knowledge after implementing an educational intervention on medication disposal [19]. Their study demonstrated that targeted educational programs could enhance public awareness of environmental risks associated with improper disposal and encourage responsible disposal behaviours. In line with this, Zulkifli et al. found that the ‘Know Your Medicine’ program effectively enhanced medication literacy among primary schoolchildren in Malaysia, reinforcing the importance of structured educational programs in improving medication-related knowledge and practices [43]. Similar education initiatives have been implemented internationally. In the United States, the Drug Enforcement Administration’s National Prescription Drug Take Back Day through educational outreach campaigns has significantly raised public awareness and participation in safe disposal practices [44]. Comparable educational intervention such as community-level educational sessions based on health behavior models have been shown to medication-related knowledge and safe disposal practices [45]. These findings underscore the effectiveness of educational initiatives in raising awareness and promoting proper medication disposal methods. Furthermore, student ambassadors model in our study not only reinforced their own knowledge but also actively engaged in educating and influencing their peers, further amplifying the initiative’s impact.

Despite overall knowledge improvement across all items, post-intervention knowledge of item 9 remained relatively low, with less than 50% of participants correctly identifying whether pressurized metered-dose inhalers (MDIs) should be discarded in the garbage. These findings are consistent with Beena et al., who reported that none of the participants in their study were aware of proper MDI disposal methods, leading to unsafe practices such as discarding, burning or burying inhalers [46]. While incineration is generally considered an environmentally friendly method to handle pharmaceutical waste, its use for MDIs poses risks of explosion and toxic gas emissions due to the presence of chlorofluorocarbons (CFCs). Hence, discarding MDIs into garbage is the better option as opposed to incineration [46]. Given this gap in knowledge, targeted education and structured take-back programs are crucial steps for minimizing unsafe MDI disposal practices.

To ensure long-term behavioural change, educational initiatives must extend beyond passive information-sharing and incorporate active engagement through peer-led programs. While structured interventions have shown promise, empowering pharmacy students to take an active role in advocating for responsible disposal practices can further enhance awareness and encourage community-wide adoption of safe disposal behaviours. By equipping future pharmacists with necessary knowledge and leadership skills, peer-led initiatives can bridge this gap, fostering a generation of healthcare professionals who are not only informed but also proactive in promoting sustainable medication disposal practices.

## Limitation

This study is subject to self-selection bias in both phases. Ambassadors in Phase 1 volunteered based on interest, which may have resulted in a cohort already motivated toward advocacy and experiential learning. Similarly, Phase 2 participants were recruited through convenience sampling, and the response rate suggests that students with greater intrinsic interest in environmental or public health issues may have been more likely to participate. Although all ambassadors received uniform training delivered through a standardised module, variation in individual engagement may have influenced the quality and delivery of the peer-led workshop. These factors should be considered when interpreting the study’s findings and when attempting replication in other educational settings.

While the findings demonstrate significant improvement in knowledge following the intervention, this study has several limitations that should be considered. Both phases involved relatively small samples (Phase 1: n = 35; Phase 2: n = 43), which may restrict statistical power and limit the variability of perspectives captured. Additionally, all participants were recruited from a single national context (Malaysia), and educational structures, disposal regulations, and cultural attitudes toward pharmaceutical waste may differ across regions. These contextual factors reduce the generalizability of the findings and suggest that results should be interpreted with caution when considering application to pharmacy curricula or public health initiatives in other countries. Future multi-centre studies involving larger and more diverse cohorts would be valuable for validating the transferability of this model across different educational and policy environments.

Phase 2 employed a one-group pretest–posttest design without a control group, meaning that the observed improvements in knowledge cannot be attributed exclusively to the workshop. Gains may also reflect factors beyond the intervention, including test familiarity or concurrent exposure to related material. In addition, several methodological considerations should be noted. First, the reflective journals are self-reported narratives and may be subject to social desirability bias, particularly as students may feel implicitly motivated to demonstrate learning, advocacy, or professional growth. Second, although the 10-item knowledge questionnaire demonstrated acceptable internal consistency, its validation among Malaysian pharmacy students remains limited, and further psychometric testing across diverse cohorts is warranted. Third, the use of a single immediate post-test captures short-term recall and may overestimate longer-term knowledge retention. Future research should therefore include larger sample sizes, multi-site cohorts, appropriate control or comparison groups, and longitudinal follow-up to evaluate both the effectiveness and sustainability of peer-led educational interventions over time. Within these constraints, we have emphasised transparent reporting and effect size estimation to aid interpretation and support future evidence synthesis.

## Conclusion

This study demonstrates a structured, peer-led experiential learning model that effectively improves pharmacy students’ knowledge and advocacy skills related to safe medication disposal. By combining hands-on engagement with peer teaching, the approach offers a practical pathway for strengthening students’ readiness to promote responsible disposal practices.

To enhance curriculum relevance, pharmacy programs may embed structured experiential components; such as community pharmacy engagement and ambassador-led workshops; into existing courses, while accreditation bodies could incorporate competencies related to medication disposal and environmental stewardship. Such integration may create sustainable opportunities for student leadership, peer-driven learning, and public health advocacy.

While the model showed meaningful improvements, the modest sample sizes and single-country context limit generalizability. Larger, multi-institutional studies are needed to assess long-term knowledge retention and to validate the applicability of this educational approach across diverse educational settings. Nonetheless, the findings offer a promising foundation for embedding peer-led experiential learning within pharmacy education and other health professions.

## Supporting information

S1 Data File De-identified pre- and post-test dataset supporting the quantitative analyses of knowledge toward return and disposal of unused medicines. (XLS)

## References

[1] Kinrys G, Gold AK, Worthington JJ, Nierenberg AA. Medication disposal practices: Increasing patient and clinician education on safe methods. J Int Med Res. 2018;46(3):927–39. doi: 10.1177/0300060517738681 29322845 PMC5972255

[2] Abrons J, Vadala T, Miller S, Cerulli J. Encouraging safe medication disposal through student pharmacist intervention. J Am Pharm Assoc (2003). 2010;50(2):169–73. doi: 10.1331/JAPhA.2010.09208 20199958

[3] Yazie TS, Siyum Z, Assefa A, Teshome AA, Belete AM, Debasu Addisu Z. Disposal practices, knowledge and attitude of adult patients visiting outpatient pharmacy services towards unused medicines in Debre Tabor, Northwest Ethiopia: a descriptive cross-sectional study. BMJ Open. 2024;14(10):e085124. doi: 10.1136/bmjopen-2024-085124 39438100 PMC11499776

[4] Toe J, Orok E, Erah P. Assessment of knowledge and disposal practices of unused and expired household medicines in a community in Liberia. Explor Res Clin Soc Pharm. 2023;12:100369. doi: 10.1016/j.rcsop.2023.100369 38058360 PMC10696106

[5] Bashaar M, Thawani V, Hassali MA, Saleem F. Disposal practices of unused and expired pharmaceuticals among general public in Kabul. BMC Public Health. 2017;17(1):45. doi: 10.1186/s12889-016-3975-z 28061902 PMC5219664

[6] Jarvis CI, Seed SM, Silva M, Sullivan KM. Educational campaign for proper medication disposal. J Am Pharm Assoc (2003). 2009;49(1):65–8. doi: 10.1331/JAPhA.2009.08032 19196599

[7] Nairat LL, Abahri NA, Hamdan YA, Abdel-Khaliq RT, Odeh SM, Abutaha S, et al. Assessment of practices and awareness regarding the disposal of unwanted pharmaceutical products among community pharmacies: a cross-sectional study in Palestine. BMC Health Serv Res. 2023;23(1):1035. doi: 10.1186/s12913-023-09888-5 37759203 PMC10537554

[8] Shakib FAF, Sadat N, Ahmed S, Nipa NY, Rahman M, Uddin MB. Unused and expired drug disposal practice and awareness among undergraduate students from pharmacy and other disciplines: Bangladesh perspective. Pharm Educ. 2022;22(1):573–83. doi: 10.46542/pe.2022.221.573583

[9] Mahnoora Z, Nandakumar UP, Joel JJ, Kolar R, Chand S. Impact of education on the knowledge, attitude and practice of disposal of expired and unused medications among pharmacy students. Ann Pharm Fr. 2023;81(4):667–73. doi: 10.1016/j.pharma.2022.12.008 36572275

[10] Akande-Sholabi W, Olaoye DQ, Adebisi YA. Drug take-back program: assessment of knowledge, practices, and barriers to safe disposal of unused medication among healthcare students in a Nigerian university. BMC Med Educ. 2023;23(1):810. doi: 10.1186/s12909-023-04788-y 37891609 PMC10605967

[11] Alhomoud FK, Alsadiq Y, Alghalawin L, Alhifany A, Alhomoud F. Pharmacy students’ knowledge and practices concerning the storing and disposal of household medication in Saudi Arabia. Curr Pharm Teach Learn. 2021;13(1):5–13. doi: 10.1016/j.cptl.2020.08.004 33131618

[12] Shuleta-Qehaja S, Kelmendi N. Pharmacy and Nursing Students’ Knowledge and Practices Concerning the Disposal of Unused and Expired Medicines in Kosovo. Pharmacy (Basel). 2022;10(6):145. doi: 10.3390/pharmacy10060145 36412821 PMC9680357

[13] Gubae K, Arega Moges T, Agegnew Wondm S, Bayafers Tamene F, Kiflu M, Aschale E, et al. Ecopharmacology: Knowledge, Attitude, and Medication Disposal Practice Among Pharmacy Students. Integr Pharm Res Pract. 2023;12:185–93. doi: 10.2147/IPRP.S428457 37901480 PMC10612519

[14] Cole JD, Ruble MJ. Medication disposal: how prepared are pharmacy students? Curr Pharm Teach Learn. 2016;8(6):871–5. doi: 10.1016/j.cptl.2016.08.024

[15] Kolb DA. Experiential learning: Experience as the source of learning and development. 2nd ed. Upper Saddle River: Pearson Education; 2015.

[16] Pierce B, van de Mortel T, Allen J, Mitchell C. The influence of near-peer teaching on undergraduate health professional students’ self-efficacy beliefs: A systematic integrative review. Nurse Educ Today. 2024;143:106377. doi: 10.1016/j.nedt.2024.106377 39208501

[17] Fraenkel JR, Wallen NE. How to design and evaluate research in education. New York: McGraw-Hill; 1990.

[18] Sim SM, Lai PSM, Tan KM, Lee HG, Sulaiman CZ. Development and Validation of the Return and Disposal of Unused Medications Questionnaire (ReDiUM) in Malaysia. Asia Pac J Public Health. 2018;30(8):737–49. doi: 10.1177/1010539518811161 30486652

[19] Lai PSM, Tan KM, Lee HG, Wong YY, Azhari Wasi NA, Sim SM. Effectiveness of an intervention to increase the knowledge, attitude, and practice regarding the return and disposal of unused medications. Malays Fam Physician. 2021;16(1):56–63. doi: 10.51866/oa1013 33948143 PMC8088745

[20] Benjamin S. Bloom. Taxonomy of educational objectives: The classification of educational goals. Handbook II: Affective domain. New York: David McKay Company; 1974.

[21] Rosenthal R. Meta-analytic procedures for social research. Newbury Park: Sage Publications; 1991.

[22] Cohen J. Statistical power analysis for the behavioral sciences. 2nd ed. Hillsdale: Lawrence Erlbaum Associates; 1988.

[23] Tringale M, Stephen G, Boylan A-M, Heneghan C. Integrating patient values and preferences in healthcare: a systematic review of qualitative evidence. BMJ Open. 2022;12(11):e067268. doi: 10.1136/bmjopen-2022-067268 36400731 PMC9677014

[24] Chen J, Wang Y. Social media use for health purposes: systematic review. J Med Internet Res. 2021;23(5):e17917. doi: 10.2196/17917 PMC8156131 33978589

[25] Urslak R, Ladhar S, Gauthier G, Sajwani S, Kanji S, Pammett R, et al. A Scoping Review of Planetary Health Education in Pharmacy Curricula. Am J Pharm Educ. 2025;89(3):101374. doi: 10.1016/j.ajpe.2025.101374 39954786

[26] Mathers A, Fan S, Austin Z. Climate change at a crossroads: Embedding environmental sustainability into the core of pharmacy education. Can Pharm J (Ott). 2023;156(2):55–9. doi: 10.1177/17151635231152882 36969305 PMC10034526

[27] Angelina L. Increasing awareness of planetary health and pharmaceutical waste disposal – FIP SustainabilityRx. 2025. Available from: https://sustainability.fip.org/blog/increasing-awareness-of-planetary-health-and-pharmaceutical-waste-disposal/

[28] United Nations. The 17 Goals: Sustainable Development. Available from: https://sdgs.un.org/goals

[29] Chit GN, Thin SM, Theeraroungchaisri A, Watcharadamrongkun S, Kittisopee T. Global patterns and determinants of household medicine storage and disposal: a systematic review and meta-analysis. J Pharm Policy Pract. 2026;19(1):2601936. doi: 10.1080/20523211.2025.2601936 41574325 PMC12821371

[30] Pharmacy Board of Malaysia. Standards for Undergraduate Pharmacy Programme 2024.

[31] Gleason BL, Peeters MJ, Resman-Targoff BH, Karr S, McBane S, Kelley K, et al. An active-learning strategies primer for achieving ability-based educational outcomes. Am J Pharm Educ. 2011;75(9):186. doi: 10.5688/ajpe759186 22171114 PMC3230347

[32] Ryan RM, Deci EL. Intrinsic and extrinsic motivations: Classic definitions and new directions. Contemp Educ Psychol. 2000;25(1):54–67. doi: 10.1006/ceps.1999.1020 10620381

[33] Hall K, Musing E, Miller DA, Tisdale JE. Experiential training for pharmacy students: time for a new approach. Can J Hosp Pharm. 2012;65(4):285–93. doi: 10.4212/cjhp.v65i4.1159 22919106 PMC3420851

[34] Frankel G, Louizos C, Austin Z. Canadian educational approaches for the advancement of pharmacy practice. Am J Pharm Educ. 2014;78(7):143. doi: 10.5688/ajpe787143 25258448 PMC4174385

[35] Ling JY, Ng PY, Shamsuddin AS, Zulkifli A, Lee KE. Medication Disposal Patterns and Practices with Awareness of Environmental Contamination Caused by Pharmaceuticals among the General Public in Malaysia. Asian Pac J Cancer Prev. 2024;25(8):2723–34. doi: 10.31557/APJCP.2024.25.8.2723 39205570 PMC11495461

[36] Huang Y-M, Chan H-Y, Lee P-I, Tang Y-W, Chiou T-W, Liu KCSC, et al. Exploration of changes in pharmacy students’ perceptions of and attitudes towards professionalism: outcome of a community pharmacy experiential learning programme in Taiwan. BMC Med Educ. 2022;22(1):195. doi: 10.1186/s12909-022-03261-6 35313880 PMC8938161

[37] Burch GF, Giambatista R, Batchelor JH, Burch JJ, Hoover JD, Heller NA. A Meta‐Analysis of the Relationship Between Experiential Learning and Learning Outcomes. Decision Sci J Innov Edu. 2019;17(3):239–73. doi: 10.1111/dsji.12188

[38] Danielson J, Craddick K, Eccles D, Kwasnik A, O’Sullivan TA. A qualitative analysis of common concerns about challenges facing pharmacy experiential education programs. Am J Pharm Educ. 2015;79(1):06. doi: 10.5688/ajpe79106 25741022 PMC4346818

[39] Wang LS, Aziz Z, Chik Z. Disposal practice and factors associated with unused medicines in Malaysia: a cross-sectional study. BMC Public Health. 2021;21(1):1695. doi: 10.1186/s12889-021-11676-x 34530791 PMC8447783

[40] Azmi Hassali M, Shakeel S. Unused and Expired Medications Disposal Practices among the General Public in Selangor, Malaysia. Pharmacy (Basel). 2020;8(4):196. doi: 10.3390/pharmacy8040196 33114172 PMC7712208

[41] Gidey MT, Birhanu AH, Tsadik AG, Welie AG, Assefa BT. Knowledge, Attitude, and Practice of Unused and Expired Medication Disposal among Patients Visiting Ayder Comprehensive Specialized Hospital. Biomed Res Int. 2020;2020:9538127. doi: 10.1155/2020/9538127 32908927 PMC7463377

[42] Yang SL, Tan SL, Goh QL, Liau SY. Utilization of Ministry of Health Medication Return Programme, Knowledge and Disposal Practice of Unused Medication in Malaysia. JPPCM. 2018;4(1):07–11. doi: 10.5530/jppcm.2018.1.3

[43] Zulkifli NW, Hamdan NEA, Md Hussin NS, Ibrahim N, Karuppannan M, Saman KM. Assessing the impact of the “Know Your Medicine” programme on medication literacy among children aged 10–12 years in Selangor, Malaysia: A pre- and post-survey intervention study. Pharm Educ. 2024;24(1):757–64. doi: 10.46542/pe.2024.241.757764

[44] Stewart H, Malinowski A, Ochs L, Jaramillo J, McCall K 3rd, Sullivan M. Inside Maine’s Medicine Cabinet: Findings From the Drug Enforcement Administration’s Medication Take-Back Events. Am J Public Health. 2015;105(1):e65–71. doi: 10.2105/AJPH.2014.302207 25393189 PMC4265938

[45] Frenzel O, Steig J, Hodges A. Assessing a Medication Safety and Disposal Educational Program using the Health Belief Model. Innov Pharm. 2023;14(3):10.24926/iip.v14i3.5546. doi: 10.24926/iip.v14i3.5546 38487380 PMC10936446

[46] Sukumara P, George D, M M, Nainan M, Thomas B. Are we handling used metered dose inhaler canisters safely? – A call for action to address an environmental hazard. IJIRM. 2019;4(1):24–6. doi: 10.18231/2581-4222.2019.0006

